# Diagnosis of Acute Myocarditis Following mRNA Vaccines against SARS-CoV-2: A Methodological Review

**DOI:** 10.3390/v15040929

**Published:** 2023-04-07

**Authors:** Marco Zuin, Emma Zimelli, Chiara Dalla Valle, Stefano Cavedon, Gianluca Rigatelli, Claudio Bilato

**Affiliations:** 1 Division of Cardiology, West Vicenza General Hospitals, Via del Parco 1, 36071 Arzignano-Vicenza, Italy; emma.zimelli@aulss8.veneto.it (E.Z.); chiara.dallavalle@aulss8.veneto.it (C.D.V.); stefano.cavedon@aulss8.veneto.it (S.C.); 2 Department of Translational Medicine, University of Ferrara, 44100 Ferrara, Italy; 3 Department of Cardiology, Ospedali Riuniti Padova Sud, 35043 Monselice, Italy; jackyheart@libero.it

**Keywords:** COVID-19, vaccines, myocarditis, SARS-CoV-2

## Abstract

The occurrence of acute myocarditis following the administration of mRNA vaccines against SARS-CoV-2 remains relatively rare, and it is associated with a very low mortality rate. The incidence varied by vaccine type, sex, and age and after the first, second, or third vaccination dose. However, the diagnosis of this condition often remains challenging. To further elucidate the relationship between myocarditis and SARS-CoV-2 mRNA vaccines, starting with two cases observed at the Cardiology Unit of the West Vicenza General Hospital located in the Veneto Region, which was among the first Italian areas hit by the COVID-19 pandemic, we performed a review of the available literature to highlight the clinical and diagnostic elements that could contribute to suspicion of myocarditis as an adverse event of SARS-CoV-2 immunization.

## 1. Introduction

Since the beginning of mass vaccination against SARS-CoV-2 through the administration of mRNA vaccines, significant reductions in both mortality and intensive care unit admissions have been observed worldwide [1,2,3]. However, after starting mass immunization, several analyses have reported potentially associated adverse reactions, including acute myocarditis [4,5,6]. Current evidence regarding the association between COVID-19 vaccines and myocarditis is largely based on published case reports and case series, as well as on observational investigations and meta-analyses [7]. Notably, according to the current international guidelines, the diagnosis of myocarditis should be based on histological, immunological, and immunohistochemical criteria, while molecular techniques are recommended to identify viral etiology [8,9]. However, few cases of acute myocarditis potentially associated with SARS-CoV-2 immunization have been diagnosed when performing an endomyocardial biopsy, leaving some doubts regarding the real incidence of this complication [4,5,6,7]. Indeed, most published reports have based the diagnosis of myocarditis on the result obtained by the cardiac magnetic resonance (MRI) [10,11,12,13,14], which could represent a valuable alternative for the non-invasive characterization of myocardial tissue. To further elucidate the relationship between myocarditis and the administration of mRNA vaccines against SARS-CoV-2, starting with two cases observed at the Cardiology Unit of the West Vicenza General Hospital located in the Veneto Region, which was among the first Italian areas hit by the COVID-19 pandemic, we reviewed the existing literature to highlight the clinical and diagnostic elements that could contribute to a suspicion of acute myocarditis as an adverse event of SARS-CoV-2 immunization.

## 2. Case 1

**Figure 1:**
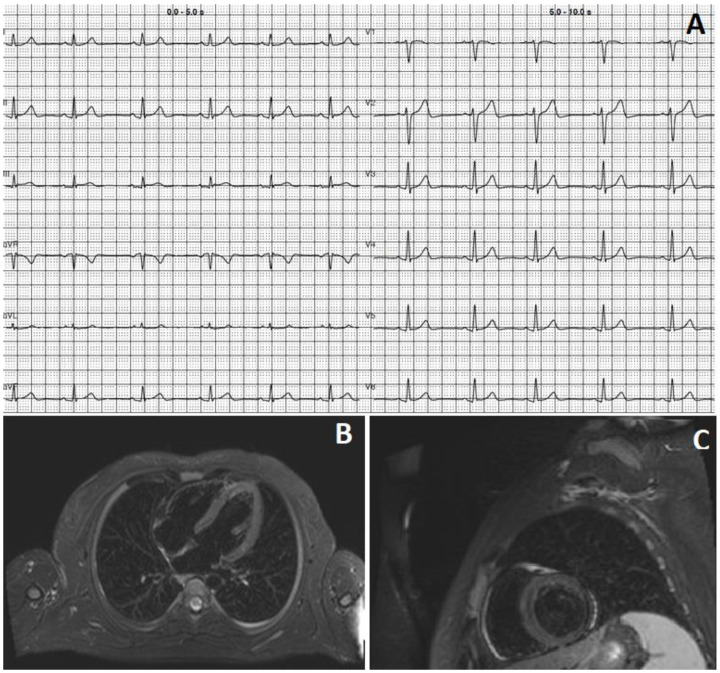
(**A**) 12-lead ECG showing mild isolated ST-segment in DIII; (**B**,**C**): Four-chamber and short-axis T2-weighted short-tau inversion recovery (T2 STIR) sequences cardiac magnetic resonance views showing patchy intramyocardial areas with late-gadolinium enhancement (LGE).

A 26-year-old man with a previous history of viral myocarditis 5 years earlier and a laparoscopic sleeve gastrectomy presented to the hospital because of pain in the left shoulder radiating to the neck. His BMI was 28.1 Kg/m^2^, with regular dietary and lifestyle habits since the gastrectomy. Five days previously, he was vaccinated for the third time (booster dose) against SARS-CoV-2 with Comirnaty^®^ (BioNTech/Pfizer, Mainz, Germany). The following day, he experienced flu-like symptoms, which quickly resolved. At admission, physical examination was unremarkable and showed normal blood pressure without signs of cardiac congestion. The electrocardiogram (ECG) revealed sinus rhythm and a mild isolated ST-segment in DIII (Figure 1, Panel A). Laboratory tests revealed increased levels of high-sensitivity troponin T (800 ng/L, n.v. < 14 ng/L) and high-sensitivity C-Reactive Protein (30 mg/L, n.v. < 1 mg/L), which further increased to 51 mg/L during the hospitalization. The transthoracic echocardiography (TTE) showed a mildly reduced left ventricular ejection fraction (LVEF) (50%) and wall motion abnormalities in the inferolateral region. The cardiac magnetic resonance (CMR) evidenced myocardial edema and patchy intramyocardial areas with late-gadolinium enhancement in the inferolateral region, suggesting acute myocarditis (Figure 1, Panels B and C). The patient was treated with a low dose of ACE inhibitors and beta-blockers. The follow-up examination after four weeks was unremarkable, while the TTE showed normal left ventricular function.

## 3. Case 2

**Figure 2:**
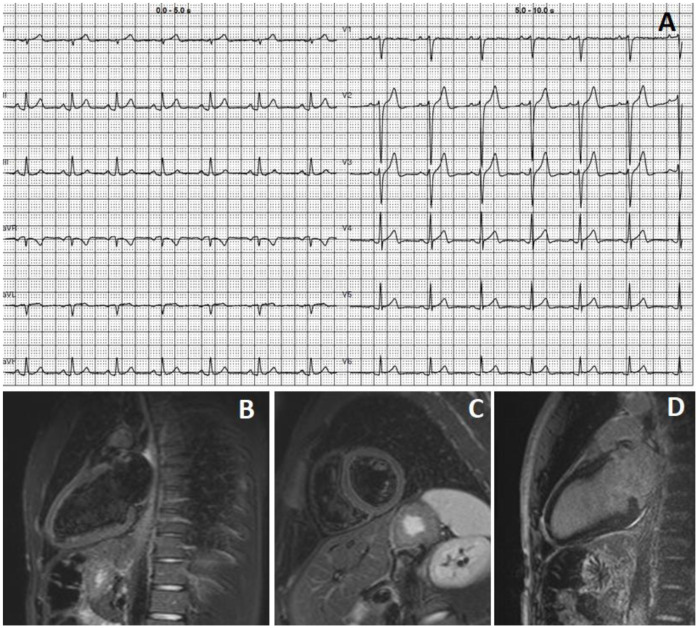
(**A**) ECG showing sinus rhythm, mild ST-segment elevation, and diphasic T waves. (**B**–**D**): T2-weighted short-tau inversion recovery (T2 STIR) sequences showing oedema of anterolateral myocardial segments with streaky late gadolinium enhancement involving the lateral left ventricular wall.

A 27-year-old man with an unremarkable previous medical history and a normal BMI (20.6 kg/m^2^), lifestyle, and dietary habits presented to the hospital because of two episodes of chest pain that occurred the day before. Specifically, three days previously, he was vaccinated for the third time (booster dose) against SARS-CoV-2 with Comirnaty^®^ (BioNTech/Pfizer). Upon admission, the patient was hemodynamically stable, while the ECG showed sinus rhythm with diphasic T-waves in anterior leads (Figure 2, Panel A). Both high-sensitivity troponin T and high-sensitivity C-reactive protein were elevated (400 ng/L, n.v. < 14 ng/L and 10 mg/L, n.v. < 1 mg/L, respectively). The TTE revealed normal left ventricular systolic function, with an LVEF of 56%. No pericardial effusion was observed. On CMR T2-weighted short-tau inversion recovery (T2 STIR), sequences revealed oedema of the anterolateral myocardial segments, while streaky late gadolinium enhancement of the lateral left ventricular wall in a non-ischemic distribution pattern was noted, corroborating a diagnosis of myocarditis according to the updated Lake Louise criteria (Figure 2, Panels B and C) [15]. The hospitalization was uneventful, and the patient was discharged at home with a low dose of ACE inhibitors and beta-blockers. In this case, four weeks after discharge, a follow-up clinical examination was unremarkable.

## 4. Pathophysiological Aspects

Currently, the exact pathophysiological mechanisms underlying the onset of acute myocarditis after the administration of the anti-COVID-19 vaccine in some patients are not yet fully understood. SARS-CoV-2 mRNA vaccines contain nucleoside-modified mRNA, which is encapsulated in lipid nanoparticles that act as delivery vehicles to transport mRNA into the cells and encode the viral spike glycoprotein of SARS-CoV-2. The mRNA promotes the synthesis of the spike protein, which in turn stimulates an adaptive immune response to identify and destroy the virus expressing the same type of spike protein. In some cases, selected RNA molecules can be immunogenic and stimulate the innate immune system, which destroys the mRNA vaccine before it reaches the target cells, preventing the production of the spike protein and the neutralizing antibodies. This process might promote the activation of an aberrant innate and acquired immune response that leads to the activation of proinflammatory cascades and immunologic pathways, triggering myocarditis [16,17,18].

## 5. Epidemiological Aspects

As demonstrated by several recent analyses, the incidence of myocarditis following SARS-CoV-2 immunization varied by the vaccine type, sex, age, and after the first, second, or third vaccination dose [19]. The overall risk of myopericarditis after mRNA vaccination against SARS-CoV-2 has been estimated to be around 22 cases per million doses [20]. Some disparities were also noted; indeed, young males, especially after the second vaccination dose, seemed to be the group at higher risk [18]. However, the reported risk of myocarditis markedly varies according to the analyzed population and the associated previous medical history [19,20,21,22]. Probably, young subjects are more prone to develop acute myocarditis after SARS-CoV-2 immunization because of a hyperimmune response; in fact, young individuals have a stronger immune response compared with older ones [23]. The observed disparity between the sexes may be explained by the potential role of hormonal differences. Previous investigations have demonstrated that testosterone may promote a more aggressive immune response by inducing CD4+ cells and inhibiting anti-inflammatory immune cells, whereas estrogen may suppress pro-inflammatory lymphocytes [24].

## 6. Comorbidities and Clinical Presentation

Recent reviews on the issue have highlighted that in patients developing myocarditis after the SARS-CoV-2 mRNA vaccine, comorbidities are present in less than half the number of cases. In most cases, the underlying disease is represented by an inflammatory or autoimmune disorder, while a previous history of myocarditis is present in only about 10% of cases [25]. However, some cardiovascular conditions, such as arterial hypertension, hypercholesterolemia, and aortic root dilation, may also act as contributing factors in the development of SARS-CoV-2-related myocarditis [26]. The onset of symptoms of myocarditis after exposure to a potential immunological trigger was shorter for anti-COVID-19 vaccine-associated cases of myocarditis than in typical myocarditis cases diagnosed after a viral illness [27,28,29]. Indeed, cases of myocarditis developed after anti-COVID-19 vaccination are usually diagnosed within a few days after vaccination. This aspect differs from the clinical presentation of typical viral myocarditis, which often has an indolent course with delayed symptoms after the trigger [19]. The symptoms of myocarditis following COVID-19 vaccination are generally not specific and occur in about half of the patients (Table 1) [27,28,29]. Notably, in most cases, the disease remains asymptomatic, and the diagnosis may be incidental when the patient undergoes a CMR because of other cardiovascular reasons. Importantly, patients experiencing anti-COVID-19 mRNA vaccination-related myocarditis also show a shorter clinical course compared with those with typical viral myocarditis [30].

## 7. Investigations

**Figure 3:**
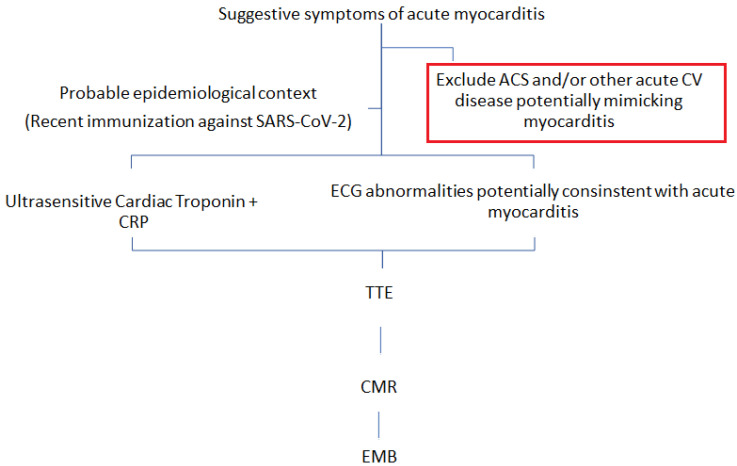
Potential flow chart for the diagnosis of acute myocarditis due to SARS-CoV-2 immunization.CV: cardiovascular disease; ACS: Acute coronary syndrome; CRP: C-reactive protein; TTE: Transthoracic echocardiogram; CMR: Cardiac magnetic resonance; EMB: Endomyocardial biopsy.

On the suspicion of anti-COVID-19 vaccination-related myocarditis, the diagnostic approach should be the same as is adopted for patients with traditional viral myocarditis (Figure 3). Blood tests have a primary role in the diagnostic management of these patients; indeed, inflammatory markers, including C-Reactive Protein and erythrocyte sedimentation rate, are usually raised, as well as the white blood count [31]. Moreover, baseline cardiac markers (e.g., troponin and N-terminal pro–B-type natriuretic peptide [NT-proBNP]) at the hospital admission are generally elevated in myocarditis because of the acute myocardial injury and the possible concomitant ventricular dilation. However, it should be noted that a negative troponin result cannot exclude myocarditis [32]. The ECG represents another test that must be performed in every patient with suspicion of acute myocarditis and, more in general, chest pain. Diffuse ST-segment elevation, with or without PR segment depression, may be observed in myocarditis or peri-myocarditis. However, as in the cases presented herein, it must be highlighted that these findings are not sensitive in detecting the disease, and their absence is not exclusionary. Some patients with acute myocarditis due to the anti-COVID-19 mRNA vaccination may have new onsets of arrhythmic events or conduction disorders, such as bundle branch block, QT prolongation, pseudo infarct pattern, premature ventricular complexes, and advanced atrioventricular blocks [33,34,35,36]. However, the confirmatory diagnosis remains histopathological. The two histopathology benchmarks currently used to diagnose myocarditis are the Dallas Criteria and the European Society of Cardiology criteria, developed in 2013, which added immunohistochemistry for the detection of CD3+ T cells and CD68+ macrophages evaluation [37]. Notably, in a non-neglectable number of patients, the histopathological findings did not meet the histological criteria for myocarditis, such as marked inflammatory cell infiltration and macrophages [38], further complicating the diagnosis. Finally, a CMR represents an alternative to an EMB, providing noninvasive tissue characterization, including inflammatory stages and patterns. Anti-COVID-19 mRNA vaccine-related myocarditis is characterized by LGE and myocardial edema [11,14,15,39,40].

## 8. Differential Diagnosis

When managing a patient with a potential SARS-CoV-2 mRNA vaccine-related myocarditis, the differential assessment of other clinical conditions with similar presentation is mandatory to avoid diagnostic errors. The potential diseases that should be excluded include acute coronary syndrome, sepsis-induced cardiomyopathy, and stress-induced cardiomyopathy. However, recent practical workflows have provided some causality algorithms that may be useful to ascertain the relationship between the occurrence of adverse events after immunization (AEFI) against SARS-CoV-2 [41,42,43]. For example, clinicians may use a four-step algorithm aimed at identifying a temporal relationship between the occurrence of the acute event and the vaccine administration and thus obtain a valid diagnosis for the referred AEFI. Then, a causality question must be formulated to exclude other potential causes, as well as the presence of pre-existing conditions before immunization that may have triggered the observed events [44].

## 9. Future Directions

In the future, further important contributions will derive from the applications of proteomics [45] and/or metabolomics [46], integrated with traditional clinical biomarkers currently applied in clinical practice [47]. Such integration will allow us to further characterize these subjects experiencing acute myocarditis after immunization. Furthermore, an important factor related to COVID-19 severity, as well as the potential onset of post-COVID-19 sequelae, such as acute myocarditis, is represented by the role of monocytes. Indeed, recent findings suggest that these cells may split into two different subpopulations: a pro-inflammatory and an anti-inflammatory subpopulation, respectively [48]. A similar pathophysiological mechanism is observed for the CD8 T and NK cells. Further data will derive from the more detailed description of the pro-inflammatory pathways as well as some specific phenotypes of inflammatory cells, such as CD8 T cells and NK cells, after immunization [49,50]. Finally, future studies will have to assess the multi-omic profiles of these patients and compare them with those observed in subjects who experienced long COVID [51,52].

## 10. Conclusions

The occurrence of acute myocarditis after immunization against SARS-CoV-2 using mRNA vaccines remains relatively rare and is associated with a very low mortality rate [16,41,42]. Indeed, the benefits of all types of vaccines approved by WHO against SARS-CoV-2 still outweigh the risks, and vaccination, if available, is highly recommended for everyone 12 years of age or older [51]. However, clinicians must be aware that myocarditis could be a potential early complication of the vaccine, which requires a prompt diagnosis and yet remains associated with a good prognosis.

## Figures and Tables

**Table 1:** Potential cardiovascular and non-cardiovascular symptoms reported as initial symptoms in patients with acute myocarditis after SARS-CoV-2 immunization.

Fever	Aspecific symptoms
Myalgia	
Headache	
Malaise	
Nausea	
Vomiting	
Diarrhoea	
Arthralgia	
Shortness of breath	Cardiovascular symptoms
Chest pain (with or without radiation)	
Palpitation	
Edema of lower limb	

## Data Availability

Not applicable.
